# Bias and Accuracy of Glomerular Filtration Rate Estimating Equations in the US: A Systematic Review and Meta-Analysis

**DOI:** 10.1001/jamanetworkopen.2024.1127

**Published:** 2024-03-05

**Authors:** Alice F. Yan, Michelle Y. Williams, Zumin Shi, Richard Oyekan, Carol Yoon, Raffick Bowen, Glenn M. Chertow

**Affiliations:** 1 Department of Research, Patient Care Services, Stanford Healthcare, Palo Alto, California; 2 Division of Primary Care and Population Health, Department of Medicine, Stanford University School of Medicine, Palo Alto, California; 3 Human Nutritition Department, College of Health Sciences, QU Health, Qatar University, Doha 2713, Qatar; 4 Department of Pathology, Stanford Healthcare, Palo Alto, California; 5 Division of Nephrology, Department of Medicine, Stanford University School of Medicine, Palo Alto, California

## Abstract

**Importance:**

There is increasing concern that continued use of a glomerular filtration rate (GFR) estimating equation adjusted for a single racial group could exacerbate chronic kidney disease-related disparities and inequalities.

**Objective:**

To assess the performance of GFR estimating equations across varied patient populations.

**Data Sources:**

PubMed, Embase, Web of Science, ClinicalTrials.gov, and Scopus databases were systematically searched from January 2012 to February 2023.

**Study Selection:**

Inclusion criteria were studies that compared measured GFR with estimated GFR in adults using established reference standards and methods. A total of 6663 studies were initially identified for screening and review.

**Data Extraction and Synthesis:**

Following Preferred Reporting Items for Systematic Reviews and Meta-Analyses (PRISMA) guidelines, 2 authors independently extracted data on studies that examined the bias and accuracy of GFR estimating equations. For each outcome, a random-effects model was used to calculate pooled estimates. Data analysis was conducted from March to December 2023.

**Main Outcomes and Measures:**

The primary outcomes were bias and accuracy of estimated GFRs in Black vs non-Black patients, as well as in individuals with chronic conditions. Bias was defined as the median difference between the measured GFR and the estimated GFR. Accuracy was assessed with P_30_ (the proportion of persons in a data set whose estimated GFR values were within 30% of measured GFR values) and measures of heterogeneity.

**Results:**

A total of 12 studies with a combined 44 721 patients were included. Significant heterogeneity was found in the bias of various GFR estimation equations. Race-corrected equations and creatinine-based equations tended to overestimate GFR in Black populations and showed mixed results in non-Black populations. For creatinine-based equations, the mean bias in subgroup analysis was 2.1 mL/min/1.73 m^2^ (95% CI, –0.2 mL/min/1.73 m^2 ^to 4.4 mL/min/1.73 m^2^) in Black persons and 1.3 mL/min/1.73 m^2^ (95% CI, 0.0 mL/min/1.73 m^2^ to 2.5 mL/min/1.73 m^2^) in non-Black persons. Equations using only cystatin C had small biases. Regarding accuracy, heterogeneity was high in both groups. The overall P_30_ was 84.5% in Black persons and 87.8% in non-Black persons. Creatinine-based equations were more accurate in non-Black persons than in Black persons. For creatinine–cystatin C equations, the P_30_ was higher in non-Black persons. There was no significant P_30_ difference in cystatin C–only equations between the 2 groups. In patients with chronic conditions, P_30_ values were generally less than 85%, and the biases varied widely.

**Conclusions and Relevance:**

This systematic review and meta-analysis of GFR estimating equations suggests that there is bias in race-based GFR estimating equations, which exacerbates kidney disease disparities. Development of a GFR equation independent of race is a crucial starting point, but not the sole solution. Addressing the disproportionate burden of kidney failure on Black individuals in the US requires an enduring, multifaceted approach that should include improving diagnostics, tackling social determinants of health, confronting systemic racism, and using effective disease prevention and management strategies.

## Key Points

**Question:**

How do glomerular filtration rate (GFR) estimating equations vary in bias and accuracy across various patient populations, and what is the association of biomarkers (eg, creatinine, cystatin C, and their combination) with the performance of these equations?

**Findings:**

In this systematic review and meta-analysis of 12 studies with a combined 44 721 patients, substantial variability was observed in GFR estimation equations. Race-based equations often overestimated GFR in Black individuals whereas serum cystatin C–based GFR estimating equations demonstrated minimal bias.

**Meaning:**

These findings suggest that creatinine-based equations are limited in their ability to estimate kidney function and underscore the need for alternative approaches such as cystatin C–based equations, as well as addressing social determinants of health and systemic racism.

## Teaser

This systematic review and meta-analysis assesses bias and accuracy in glomerular filtration rate estimating equations across various adult populations in the US.

## Introduction

Although access to dialysis care for patients with end stage kidney disease who qualify for Medicare is guaranteed by law, there are substantial and persistent racial disparities in chronic kidney disease (CKD) burden, outcomes, and care. Notably, Black patients are disproportionately affected by CKD and its progression to kidney failure yet are less likely to receive kidney replacement treatments or be placed preemptively on a transplant waitlist compared with White patients.^1,2^ In light of these disparities, timely identification and diagnosis of CKD is essential to mitigate its progression and associated complications.

Glomerular filtration rate (GFR) is regarded as the most reliable indicator of both normal and impaired kidney function. Measuring GFR can be accomplished using inulin or by assessing the clearance of exogenous filtration markers, such as technetium Tc-99m diethylenetriamine pentaacetic acid, chromium 51-ethylenediamine tetraacetic acid, or iohexol.^3^ However, these benchmark methods for measured GFR (mGFR) are not commonly used in clinical practice due to their complexity and cost. Hence, the estimated glomerular filtration rate (eGFR), which is used in eGFR equations, is a practical tool for assessing kidney function and identifying CKD. Current eGFR equations are integral for determining the burden of kidney disease at the population and patient level; however, they are limited by (1) poor individual-level (patient) predictions,^4,5^ (2) imprecise population predictions for key CKD-related conditions (eg, diabetes^6^), (3) more pronounced bias at GFR levels greater than 60 mL/min/1.73 m^2^, and (4) need for race correction with serum creatinine–based GFR estimating equations for Black individuals.^7,8,9,10^

The development of eGFR equations has evolved over time, with recent efforts aiming to address their limitations. Notably, in 2021, a workgroup assembled by the National Kidney Foundation and American Society of Nephrology recommended an updated CKD Epidemiology Collaboration (CKD-EPI) creatinine equation that was refit without a race coefficient.^11^ A recent review^12^ indicated that the inclusion of Black race correction in eGFR equations yielded no clinical, statistical, or analytical benefit toward clinical diagnoses and treatment, and may contribute to health care inequities and social harms for Black individuals in the US. In this systematic review and meta-analysis, we examined the performance of commonly used eGFR equations in terms of bias and accuracy across various patient populations. Additionally, we assessed the association of different biomarkers (creatinine, cystatin C, and their combination) with the performance of these equations. The ultimate goal was to understand how these equations inform clinical decision-making within US health care settings.

## Methods

### Data Source and Literature Search Strategy

This study followed the Preferred Reporting Items for Systematic Reviews and Meta-Analyses (PRISMA) checklist and reporting guideline. We conducted a systematic review to summarize the existing studies.^13^ The search was conducted in PubMed, Embase, Web of Science, clinicaltrials.gov, and Scopus databases to locate studies that simultaneously obtained mGFR and eGFR in adults. Studies included were those that independently derived and validated eGFR equations using reference methods with direct measurements of creatinine, cystatin C, or both in US populations. The search was augmented by scanning Grey Literature Network Services and the National Kidney Foundation Kidney Disease Outcomes Quality Initiative practice guidelines. We included studies that tested the performance of the eGFR equations in various patient groups, including Black patients and non-Black patients, and those with chronic conditions including obesity, diabetes, and/or hypertension, along with potential kidney donors, and other healthy individuals.

### Study Selection: Inclusion and Exclusion Criteria

Studies met inclusion criteria if they reported the recommended reference standards and methods, recruited only adults (≥18 years) from the US in their derivation cohort or study participants, and were published from January 2012 to February 2023. We excluded studies of patients with acute kidney diseases, pregnant individuals, individuals younger than 18 years, or critically ill patients in whom serum creatinine concentrations were in a nonsteady state.^14^ We also excluded studies in patients treated for rare conditions, case series, studies with fewer than 40 subjects, opinion pieces, and other reviews.

Two independent reviewers (R.O. and J.G.P.) screened all the results to determine eligibility for this review. A third reviewer (C.Y.) independently resolved disagreements. Detailed search terms including key words, and medical subject headings, and steps are given in the eTable in Supplement 1.

### Data Extraction and Quality Assessment

Data screening and extraction involved a 2-phase procedure: a title and abstract review screening and a full-text review. Both phases were blinded (ie, each reviewer could not see the decision of others) to prevent reviewer bias and were conducted in Covidence, a web-based systematic review tool (Veritas Health Innovation).^15^ Two independent reviewers (R.O. and A.Y.) extracted the data.

For selected studies, we extracted the following information: author and publication year, study design, study population, race (categorized as Black or non-Black), how race was defined, methods used for measuring GFR, assessments of social determinants of health (SDOH [eg, education status and socioeconomic status]), mean (95% CI) bias, sample size, and accuracy. In regards to race and ethnicity categorizations, each study had its own methods of categorization and definition of non-Black. For each data set that a study reported, we examined whether filtrate assays were standardized to body surface area and whether they were calibrated to the National Institute of Standards and Technology Isotope Dilution Mass Spectrometry (IDMS) standards. All studies included for the review and analyses were referenced to IDMS.

### Data Synthesis and Statistical Analysis

We evaluated the performance of eGFR equations based on 2 key metrics: bias and accuracy. Bias, in the context of kidney function assessment, was defined as the median difference between the mGFR and the eGFR, calculated in milliliters per minute per 1.73 m^2^ and accompanied by its 95% CI. This bias (expressed as mGFR − eGFR) reveals the systematic deviation of the eGFR in estimating kidney function as measured. A positive value of bias indicates that the eGFR tends to underestimate measured kidney function, while a negative value of bias indicates that the eGFR tends to overestimate measured kidney function. Accuracy was assessed by P_30_, the proportion of persons in a data set whose eGFR values are within 30% of measured GFR values; it is the conventional metric for assessing the accuracy of eGFR equations.^7^ P_30_ values of 90% or greater indicate high accuracy.^16^ Our analysis focused on studies that examined the performance of eGFR equations in 2 areas: (1) bias and accuracy comparing Black and non-Black participants and (2) bias and accuracy in subgroups with chronic conditions (eg, obesity, diabetes, and hypertension), as well as potential kidney donors and other healthy individuals. We summarized the results using the CKD-EPI eGFR equations (serum creatinine [cr], cystatin C [cys]), and the combined cr-cys).

We conducted meta-analyses to compare bias and accuracy of eGFR using the CKD-EPI equations with mGFR. A random-effects model was used to calculate pooled estimates of bias and accuracy, along with their 95% CIs, and *P* values. Statistically significant results were defined as a 2-sided *P* < .05. To account for heterogeneity, we performed random-effects meta-regression. Heterogeneity was assessed using the *I^2^* statistic,^17^ classified as low (<25%), moderate (25%-75%), or high (>75%). We conducted subgroup analyses to explore potential sources of heterogeneity. Publication bias was evaluated using funnel plot analysis. We conducted all statistical analyses using Stata version 18.0 (StataCorp). Data analysis was conducted from March to December 2023.

## Results

### Summary of Studies

The flow diagram (eFigure 1 in Supplement 1) summarizes the study selection process. Out of the initial pool of 6663 studies, 12 studies^18,19,20,21,22,23,24,25,26,27,28,29^ with a total of 44 721 participants met our inclusion criteria. Of the 12 studies, 6 studies with 23 validations^18,19,20,21,22,23^ specifically examined bias and accuracy in eGFR equations (eGFR_cr_, eGFR_cys_, and eGFR_cr-cys_) between Black and non-Black populations. The remaining 6 studies^24,25,26,27,28,29^ included bias and accuracy of eGFR equations in patients with chronic conditions (eg, obesity, diabetes, and hypertension), as well as potential kidney donors, and other healthy individuals. None of the studies measured SDOH. The characteristics of the included studies are summarized in the Table.

**Table.** The Characteristics of Included Studies That Examined Bias and Accuracy of GFR Estimation Equations^a^

Source	Country	Study design and data source	Population	Black participants, No./ Total No. (%)	Non-black participants, No./Total No. (%)	How race was defined	Social determinants of health measure	measured GFR	Outcomes reported
**Analysis for Black and Non-Black populations**									
Inker et al,^18^ 2018	US	Ancillary study of the Multi-Ethnic Study of Atherosclerosis-Kidney cohort	Community-based sample of 294 older individuals at 1 site	139/294 (47.3); 99/139 (71.2) had hypertension and 47/139 (33.8) had diabetes	155/294 White (52.7)	Self-reported	No	Iohexol	Bias and P_30_
Inker et al,^19^ 2021	US	Cross-sectional; used the CKD-EPI cystatin C and creatinine-cystatin C external development and validation data set	Pooled data from research studies and clinical populations in which GFR was measured; 12 studies used for validation	579/4050 (14.3); 171/512 (33.4) had diabetes and 167/579 (28.9) had BMI ≥ 30^b^	3471/4050 (85.7)^c^	Self-reported	No	Exogenous filtration markers (eg, iothalamate, iohexol, ^51^Cr-EDTA, or 125I-iothalamate)	Bias and P_30_
Meeusen et al,^20^ 2022	US	Cross-sectional; used data from the Mayo Clinic	Outpatients with and without kidney disease	852/33 889 (2.5)	33 037 (97.5)^c^	Self-reported	No	Iothalamate	Bias and P_30_
Rocha et al,^21^ 2020	Brazil	Cross-sectional	Patients with CKD	61/100 (61.0) African Brazilian^d^	39/100 White (39.0)	Investigator-assigned	No	^51^Cr-EDTA	Bias and P_30_
Goodson et al,^22^2022	US	Cross-sectional	Potential live kidney donors evaluated at the University of California, Davis Medical Center between October 2016 and December 2020	37/637 (5.8)	328/637 White (51.5); 86/637 Asian (13.5); 186;637 Hispanic (29.2)^e^	Self-reported	No	Iohexol	Bias and P_30_
Hsu et al,^23^ 2021	US	Cross-sectional; used data from the CRIC cohort	Large national cohort	458/1248 (36.7)^f^	603/790 White; (76.3) 104/790 unknown or not reported (13.2); 60/790 Asian (7.6); 10/790 multiracial (1.3); 8/790 American Indian or Alaska Native (1.0); 5/790 Native Hawaiian or Other Pacific Islander (0.6)	Self-reported	No	125I-iothalamate	Bias and P_30_
**Analysis for patients with chronic conditions**									
Aggarwal et al,^24^2012	US	Cross-sectional	Potential kidney donors; 49/164 (29.9) had class I obesity (BMI, 30-35) and 32/164 (19.5%) had class II obesity (BMI>35)^b^	71/164 (43.3)	43/164 Hispanic (26.2); 38/164 White (23.1); 12/164 other race or ethnicity (7.3)	Self-reported	No	^99m^Tc-DTPA	Bias
Anderson et al,^25^ 2012	US	Cross-sectional; used data from the CRIC cohort	Adults from 7 metropolitan areas with kidney insufficiency in GFR subcohort	534/1433 (37.3)	46/50 White (92.0); 1/50 Hispanic (2.0); 2/50 other race or ethnicity (4.0); 1 unavailable (2.0)	Self-reported	No	125I-iothalamate	Bias and P_30_
Fan et al,^26^ 2014	US	Cross-sectional; used data from the CKD-EPI cystatin C and creatinine-cystatin C external validation data set	Patients with varying ages and BMI; 594/1119 (53.1) had diabetes^b^	30/1119 (2.7)	1089/1119 White (97.3)	Self-reported	No	125I-iothalamate	Bias and P_30_
Hingorani et al,^27^ 2015	US	Prospective cohort	Patients undergoing a hematopoietic cell transplant at the Fred Hutchinson Cancer Research Center from 2009 to 2013	Not reported	46/50 White (92.0); 1/50 Hispanic (2.0); 2/50 other race or ethnicity (4.0); 1 unavailable (2.0)	Not reported	No	Iohexol	Bias
Keddis et al,^28^ 2016	US	Cross-sectional; data used from Mayo Clinic	Cohort of kidney transplant recipients >1 y after transplant	Not reported	976/1139 (85.6) White	Not reported	No	125I-iothalamate	Bias
Guebre-Egziabher et al,^29^ 2019	France	Retrospective	Participants with obesity (BMI ≥ 35 )^b^	Not reported	Not reported; 363/706 (5.4) had diabetes and 484/706 (68.6) had hypertension	Not reported	No	Iohexol and inulin clearance	Bias and P_30_

Abbreviations: BMI, body mass index; ^51^Cr-EDTA, chromium 51-ethylenediamine tetraacetic acid; CKD-EPI, Chronic Kidney Disease Epidemiology Collaboration; CRIC, chronic renal insufficiency; GFR, glomerular filtration rate; P_30_, the proportion of persons in a data set whose estimated GFR values were within 30% of measured GFR values; ^99m^Tc-DTPA, Tc-99m diethylenetriamine pentaacetic acid.

^a^ GFR equations included serum creatinine–based equations, cystatin C–based equations, and combination serum creatinine and cystatin C–based equations.

^b^ BMI was calculated as weight in kilograms divided by height in meters squared.

^c^ No breakdown of the categories that comprise non-Black was available.

^d^ African Brazilians included 27 Black individuals and 34 multiracial (mixed-race) individuals.

^e^ We specifically extracted data from White participants to represent the non-Black group in this study. Although the study presented a detailed breakdown across Asian, Hispanic, Black, and White groups, for the purposes of our study, non-Black strictly refers to the White cohort, consisting of 328 participants.

^f^ Identified as Black or Black and multiracial.

### Bias Comparing Black and Non-Black Participants in Meta-Analysis

**Figure 1.**
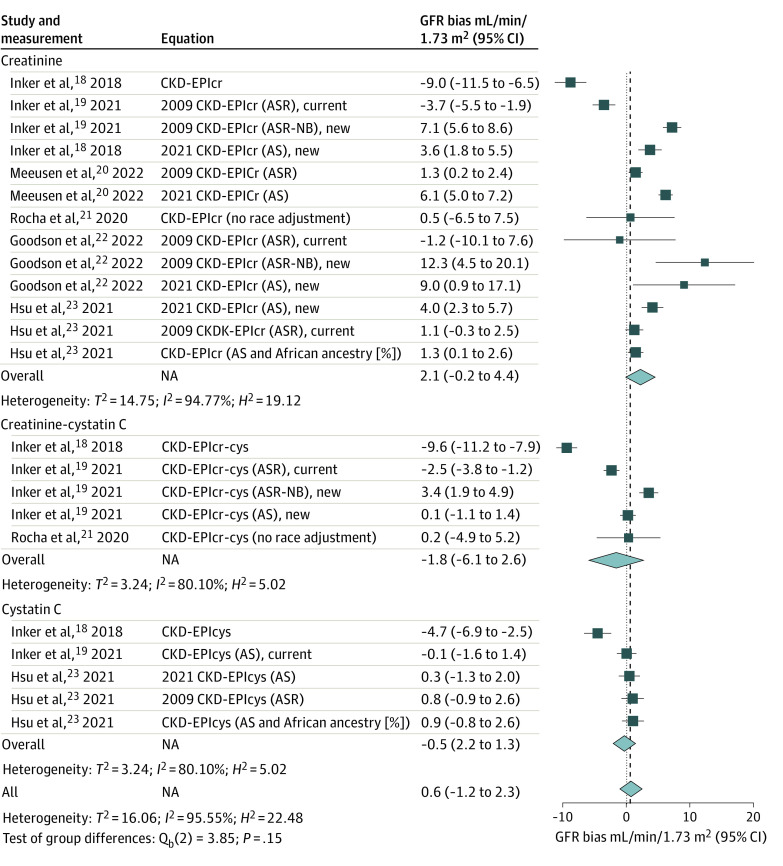
Bias in Glomerular Filtration Rate (GFR) Estimation Equations for Black Participants in Meta-Analysis The Chronic Kidney Disease Epidemiology Collaboration (CKD-EPI) GFR estimating equations (eGFR) are referred to by the filtration marker or markers (serum creatinine [cr], cystatin C [cys], and the combined cr-cys) and the demographic factors (age, sex, and race [ASR], or age and sex [AS]) that were used in their development. ASR-Non-Black (NB) refers to ASR equations that were fit with a race term but in which the Black race coefficient was removed for computing of eGFR. Bias was defined as the median difference between the measured GFR (mGFR) and the eGFR, calculated in milliliters per minute per 1.73 m^2^ (mGFR − eGFR) and accompanied by its 95% CI. A positive value of bias indicates that the eGFR tends to underestimate actual kidney function, while a negative bias value suggests an overestimation by eGFR. NA indicates not applicable.

**Figure 2.**
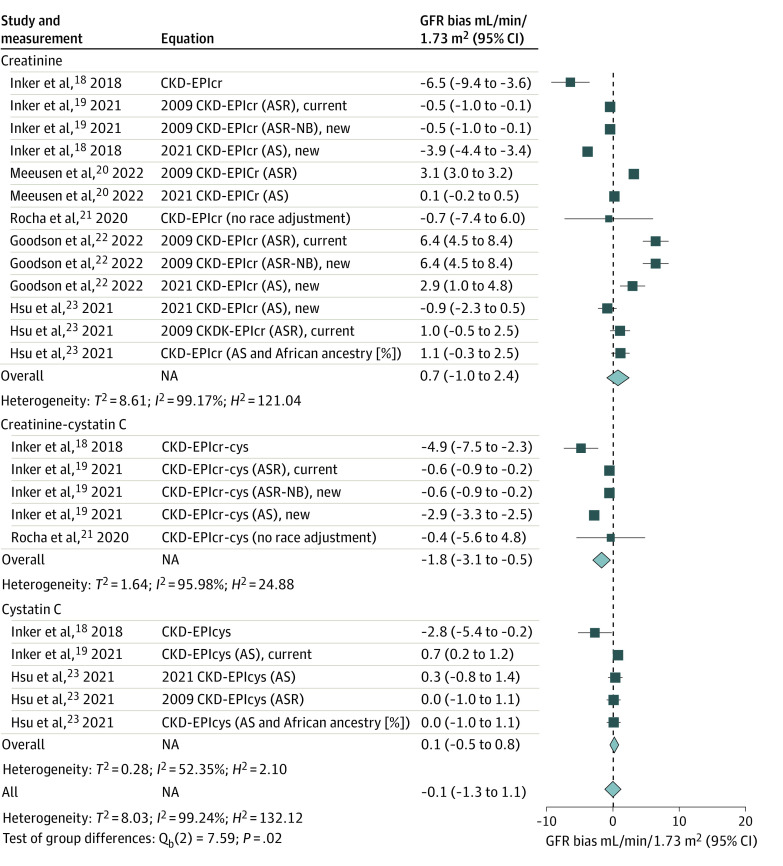
Bias in Glomerular Filtration Rate (GFR) Estimation Equations for Non-Black Participants in Meta-Analysis The Chronic Kidney Disease Epidemiology Collaboration (CKD-EPI) GFR estimating equations (eGFR) are referred to by the filtration marker or markers (serum creatinine [cr], cystatin C [cys], and the combined cr-cys) and the demographic factors (age, sex, and race [ASR], or age and sex [AS]) that were used in their development. ASR-Non-Black (NB) refers to ASR equations that were fit with a race term but in which the Black race coefficient was removed for computing of eGFR. Bias was defined as the median difference between the measured GFR (mGFR) and the eGFR, calculated in milliliters per minute per 1.73 m^2^ (mGFR − eGFR) and accompanied by its 95% CI. A positive value of bias indicates that the eGFR tends to underestimate actual kidney function, while a negative bias value suggests an overestimation by eGFR. Non-Black participants included participants who identified as Asian, Hispanic, White, and multiracial. NA indicates not applicabe.

Substantial heterogeneity of the bias was observed among different eGFR equations in Black persons and non-Black persons (Figure 1and Figure 2). Among Black persons, of the 23 validations from the 6 studies comparing the performance of eGFR_cr_, eGFR_cys_, and eGFR_cr-cys_ equations relative to mGFR,^18,19,20,21,22,23^ 5 equations from 2 studies^18,19^ overestimated GFR while 9 equations from 4 studies^19,20,22,23^ underestimated GFR (Figure 1). The race-specific, creatinine-based equations tended to overestimate GFR in Black persons. The highest overestimate was found for CKD-EPI_cr-cys_^18^ (mean bias, –9.6 mL/min/1.73 m^2^; 95% CI, –7.9 mL/min/1.73 m^2^ to –11.2 mL/min/1.73 m^2^). In Goodson et al,^22^ the 2009 CKD-EPI_cr _ equation for age, sex, and race (ASR)–non-Black, (ASR-NB; ie, an ASR equation that was fit with a race term but in which the Black race coefficient was removed for computing of eGFR) had the highest underestimation of GFR (mean bias, 12.3 mL/min/1.73 m^2^; 95% CI, 4.5 mL/min/1.73 m^2 ^to 20.1 mL/min/1.73 m^2^).

In non-Black persons, 9 equations from 2 studies^18,19^overestimated and 5 equations from 3 studies^19,20,22^ underestimated GFR (Figure 2). The highest overestimate was found for the CKD-EPI_cr_ equation^18^ (mean bias, –6.5 mL/min/1.73 m^2^; 95% CI, –3.6 mL/min/1.73 m^2^ to –9.4 mL/min/1.73 m^2^) while the highest underestimates of GFR were the 2009 CKD-EPI_cr_ (ASR-NB)^22^ and 2009 CKD-EPI_cr_ (ASR)^22^ equations (both had a mean bias of 6.4 mL/min/1.73 m^2^; 95% CI, 4.5 mL/min/1.73 m^2^ to 8.4 mL/min/1.73 m^2^).

**Figure 3.**
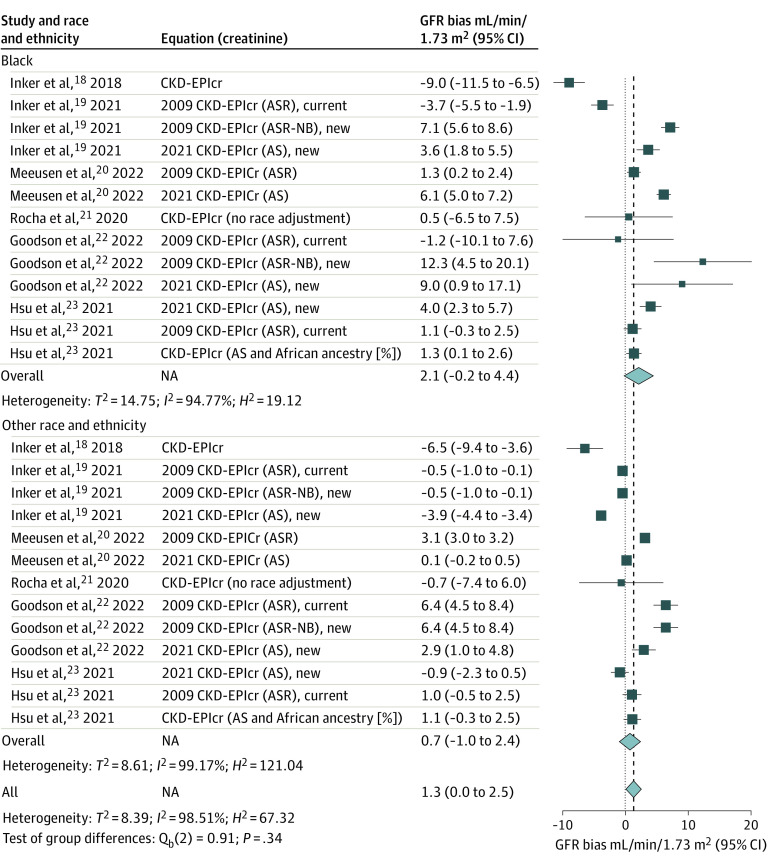
Bias in Glomerular Filtration Rate (GFR) Estimation for Creatine-Based Equations in Subgroup Analysis This figure shows Chronic Kidney Disease Epidemiology Collaboration (CKD-EPI) serum creatinine (cr)-based GFR estimating equations (eGFR) and the demographic factors (age, sex, and race [ASR], or age and sex [AS]) that were used in their development. ASR-Non-Black (NB) refers to ASR equations that were fit with a race term but in which the Black race coefficient was removed for computing of eGFR. Bias was defined as the median difference between the measured GFR (mGFR) and eGFR, calculated in milliliters per minute per 1.73 m^2^ (mGFR − eGFR) and accompanied by its 95% CI. A positive value of bias indicates that the eGFR tends to underestimate actual kidney function, while a negative bias value suggests an overestimation by eGFR. Non-Black participants included participants who identified as Asian, Hispanic, White, and multiracial. NA indicates not applicable.

Figure 3 shows the bias in eGFR for creatinine-based equations in subgroup analysis. The analysis included 13 validations for both Black and non-Black persons.^18,19,20,21,22,23^ The overall mean bias was 2.1 mL/min/1.73 m^2 ^(95% CI, –0.2 mL/min/1.73 m^2 ^to 4.4 mL/min/1.73 m^2^) in Black participants and 1.3 mL/min/1.73 m^2^ (95%, CI, 0.0 mL/min/1.73 m^2^ to 2.5 mL/min/1.73 m^2^) in non-Black participants. The studies had high heterogeneity in both groups. The variation of the bias was substantial for the Black participants, ranging from an overestimation of 9.0 mL/min/1.73 m^2^ to an underestimation of 12.3 mL/min/1.73 m^2^. Similarly, in non-Black participants, the bias varied from an overestimation of 6.5 mL/min/1.73 m^2^ to an underestimation of 6.4 mL/min/1.73 m^2^.

**Figure 4.**
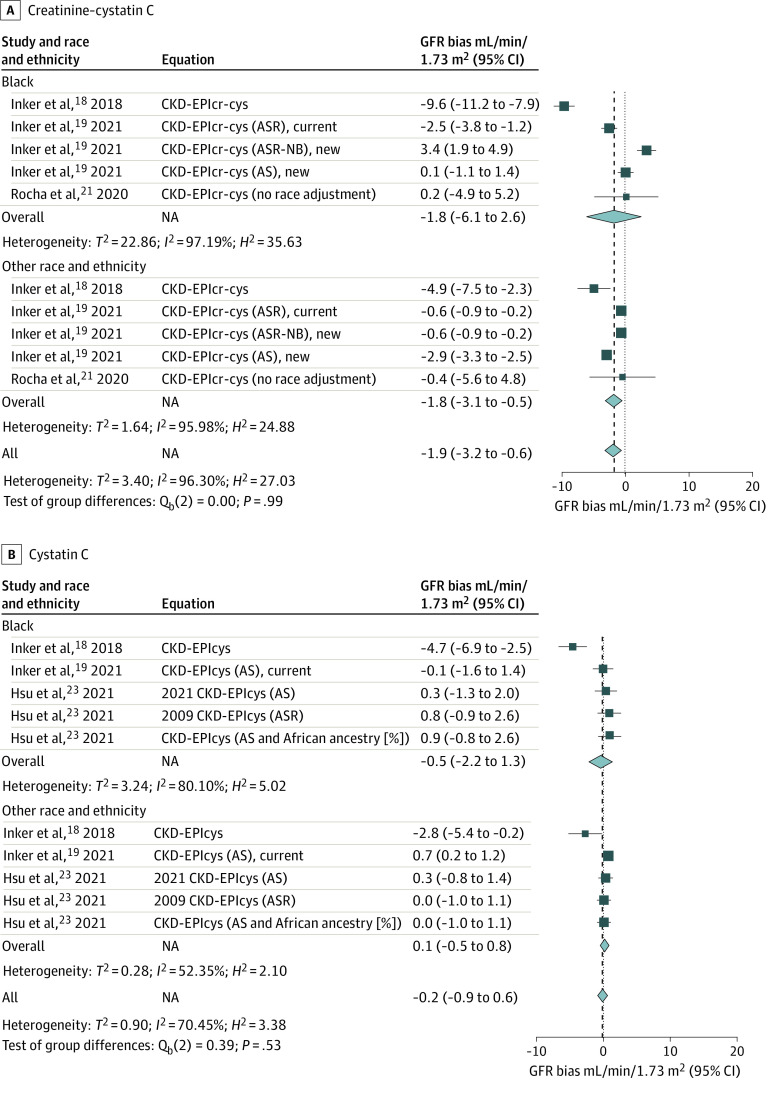
Bias in Glomerular Filtration Rate (GFR) Estimation for Creatinine-Cystatin C Combination Equations and Cystatin C Alone Equations in Subgroup Analysis The Chronic Kidney Disease Epidemiology Collaboration (CKD-EPI) GFR estimating equations are referred to by the filtration marker or markers including the combined serum cretine and cystatin C (cr-cys [A]), cystatin C alone (B), and the demographic factors (age, sex, and race [ASR], or age and sex [AS] that were used in their development). ASR-Non-Black (NB) refers to ASR equations that were fit with a race term but in which the Black race coefficient was removed for computing of estimated GFR (eGFR). Bias was defined as the median difference between the measured GFR (mGFR) and the eGFR, calculated in milliliters per minute per 1.73 m^2^ (mGFR − eGFR) and accompanied by its 95% CI. A positive value of bias indicates that the eGFR tends to underestimate actual kidney function, while a negative bias value suggests an overestimation by eGFR. Non-Black participants included participants who identified as Asian, Hispanic, White, and multiracial. NA indicates not applicable.

Figure 4A shows the bias in eGFR using creatinine-cystatin C–based equations. The overall mean (SD) bias was –1.8 mL/min/1.73 m^2^ (95% CI, –6.1 mL/min/1.73 m^2 ^to 2.6 mL/min/1.73 m^2^) in Black participants and −1.8 mL/min/1.73 m^2^ (95% CI, –3.1 mL/min/1.73 m^2^ to –0.5 mL/min/1.73 m^2^) in non-Black participants. Among the 5 validations, 2 overestimated GFR^18,19^and 1 underestimated GFR^19^ in Black persons. In non-Black persons, 4 validations overestimated GFR.^18,19^ The highest overestimations of bias were found in the CKD-EPI_cr-sys_ equation in both Black participants (9.6 mL/min/1.73 m^2^) and non-Black participants (4.9 mL/min/1.73 m^2^). The creatinine-cystatin C–based equation with age and sex alone had smaller bias in Black participants, but overestimated mGFR in non-Black participants.^19^

The bias in eGFR in equations based on cystatin C alone was small (Figure 4B). The overall mean bias was –0.5 mL/min/1.73 m^2^ (95% CI, –2.2 mL/min/1.73 m^2^ to 1.3 mL/min/1.73 m^2^) in Black participants and 0.1 mL/min/1.73 m^2^ (95% CI, –0.5 mL/min/1.73 m^2^ to 0.8 mL/min/1.73 m^2^) in non-Black participants. In Black participants, the CKD-EPI_cys_ equation overestimated GFR by 4.7 mL/min/1.73 m^2^ (95% CI, –6.9 mL/min/1.73 m^2^ to –2.5 mL/min/1.73 m^2^ ) in 1 study.^18^ In non-Black participants, the highest overestimation was found for the CKD-EPI_cys_ equation^18^ (–2.8 mL/min/1.73 m^2^; 95% CI –5.4 mL/min/1.73 m^2^ to –0.2 mL/min/1.73 m^2^). Equations with cystatin C, age, and sex alone had minimal bias in both Black and non-Black participants.^19,23^There was no significant difference in overall bias between 2 groups in all the subgroup analyses.

### Accuracy Comparing Black and Non-Black Participants in Meta-Analysis

In the analysis of 23 validations from 6 studies,^18,19,20,21,22,23^ the accuracy (P_30_) of eGFR ranged from 68.7%^20^ to 91.9%^22^ for the 2021 CKD-EPI_cr_ (age and sex) equation in Black persons (eFigure 2 in Supplement 1). In non-Black participants, P_30_ varied from 78.6%^20^ to 97.0%.^22^ There was high heterogeneity in both groups (eFigure 3 in Supplement 1). The overall P_30_ was 84.5% in Black participants and 87.8% in non-Black participants. Notably, 12 validations in Black participants and 14 in non-Black participants reported P_30_ greater than 85%.

In studies using equations based solely on creatinine,^18,19,20,21,22,23^ the overall P_30_ was 82.6% for Black participants and 87.2% for non-Black participants (eFigure 4 in Supplement 1). Overall, these equations demonstrated better accuracy in non-Black participants compared with Black participants. Three equations reported a P_30 _greater than 95% in non-Black participants, whereas only 1 equation reported a P_30_ greater than 90% in Black participants.^22^

In studies using creatinine-cystatin C–based equations,^18,19,21^ there was a significant difference in P_30_ between Black and non-Black persons (eFigure 5 in Supplement 1). The overall P_30_ was 88.1% for Black persons and 92.1% for non-Black persons. In non-Black persons, no studies reported a P_30_ less than 85%. In contrast, among Black individuals, 2 studies^18,21^ reported a P_30_ less than 85%.

In studies using cystatin C–based equations,^18,19,23^ there was no significant difference in P_30_ between Black and non-Black persons (eFigure 6 in Supplement 1). The overall P_30_ was 85.3% for Black persons and 85.7% for non-Black persons. However, no studies had a P_30_ greater than 90%.

### Accuracy and Bias in Subgroups With Chronic Conditions

In participants with chronic conditions, P_30_ was less than 85% in more than one-half of the validations (12 of 21 validations) from 3 studies^25,26,29^(eFigure 7 in Supplement 1). There was high heterogeneity in P_30_. Bias ranged from an overestimation^24^ of 19.2 mL/min/1.73 m^2 ^to an underestimation^27^ of 15.6 mL/min/1.73 m^2^ (eFigure 8 in Supplement 1).

## Discussion

This systematic review and meta-analysis analyzed 12 studies^18,19,20,21,22,23,24,25,26,27,28,29^ that used new and established regression equations to estimate GFR. In this study, substantial heterogeneity was found in the bias of different eGFR equations. Creatinine-based equations generally overestimated GFR in Black persons and showed mixed results in non-Black persons. The mean bias in subgroup analysis was 2.1 mL/min/1.73 m^2^ in Black persons and 1.3 mL/min/1.73 m^2^ in non-Black persons. Equations using cystatin C alone had small biases. Regarding accuracy, heterogeneity was high in both groups. The overall P_30_ was 84.5% in Black and 87.8% in non-Black persons. Creatinine-based equations were more accurate in non-Black persons than in Black persons. For creatinine-cystatin C equations, the P_30_ was higher for non-Black persons. There was no significant P_30_ difference in cystatin C only equations between the 2 groups, but none exceeded 90%. In patients with chronic conditions, P_30_ was generally less than 85%, with high heterogeneity and a wide range of biases.

This review raises 2 critically important points previously overlooked by most authors addressing these issues. First, while the equations are generally useful for the assessment of kidney disease across the population, their accuracy for clinical decision-making in individual patients remains disappointing. For example, the CKD-EPI equations consistently reported higher accuracies, except for patients with diabetes and severe obesity.^24,30^ This finding suggests that CKD-EPI equation may not perform well in these patients. It is important to note that plasma glucose can interfere with the measurement of creatinine using the Jaffe reaction.^31^ Therefore, it is a plausible hypothesis that diabetes-related factors, such as degree of glycemic control or pharmacological effects, could cause analytical interference with the detection of creatinine. The enzymatic creatinine methods, despite higher cost, provide increased accuracy and are less susceptible to interferences compared to the Jaffe method.^32^ Similarly, eGFR is limited in patients with severe (grade 2) obesity (body mass index [BMI] ≥35 [calculated as weight in kilograms divided by height in meters squared]) and morbid obesity (grade 3; BMI≥40),^29^ as our review documented higher bias with a BMI of 35 or greater.^24^

Second, our findings suggest that the bias and inaccuracy in serum creatinine–based eGFR equations is of similar or larger magnitude than any differences related to inclusion or noninclusion of a designated race term. Miller et al^33^ and Sehgal et al^34^ argued that uncertainty in eGFR is much larger than the race adjustment term and that eGFR, whether adjusted for race or not, provides only a rough measure of kidney function. Eliminating the Black vs non-Black race term from eGFR equations will minimize racial bias in CKD diagnosis, given that substantial racial disparities in CKD diagnosis are well-documented.^1,2^ Individuals from marginalized communities face a substantial increased risk of kidney failure, with Black persons having a 2.6 times higher risk than White persons.^35^ Moreover, research indicates that Black patients with CKD experience faster disease progression.^36^ Our results suggest that serum creatinine–based eGFR equations are particularly biased among patients with type 2 diabetes, hypertension, and obesity,^10,24,26,30^ conditions which disproportionately affect Black patients.

Racial disparities in CKD affecting Black patients stem not only from CKD risk factors but also from the root causes of these disparities, such as SDOH and systematic racism. Similar to other chronic diseases, racial disparities in CKD incidence, progression, and mortality are likely associated with factors such as neighborhood segregation,^37^ lower socioeconomic status or poverty,^38,39^ unstable housing,^39^ perceived racial discrimination,^40^ food insecurity,^41^ inadequate control of disease risk factors,^42,43^ and systemically racist and discriminatory policies and practices that limit individuals’ access to CKD care.^42^ None of the studies in our systematic review included measures of SDOH, which may have contributed to the variance observed. It is important to acknowledge that while we can measure the bias in eGFR equations, the harm caused to Black persons by this practice over the past 2 decades should be considered within the broader context of the substantial disparities they face. Addressing the root causes of these disparities is an urgent priority.

In addition, the study points out the shortcomings of relying on a single creatinine-based equation for estimating kidney function, indicating that that a one-size-fits-all (or universal) approach is not effective across the diverse range of GFR seen in clinical settings. It suggests that it is improbable for a single biomarker to adequately represent the complexity of kidney function, or specifically the aspect described as glomerular filtration.

Developing accurate predictions of GFR with race-free equations has proved challenging in clinical practice. Inker et al^19^ found that the same CKD-EPI eGFR_cr_ equation refitted without race had a similar percent agreement between eGFR and mGFR within CKD stages but retained modest statistical bias. Conversely, Hsu et al^23^ found that excluding Black race from serum creatinine-derived eGFR equations yielded larger bias and poorer accuracy. They concluded that eliminating race from these equations introduced a systematic misclassification, which persisted even when accounting for various non-GFR determinants of serum creatinine concentration.^23^ In contrast, designated race was not associated with the predictive accuracy of eGFR in equations based on cystatin C. Both Inker et al^19^ and Hsu et al^23^ suggested the promise of cystatin C for more accurate and uniform GFR prediction without race-based adjustments. This approach may help avoid potential race-based disparities in CKD diagnoses.

### Limitations

This systematic review and meta-analysis had limitations. First, it adhered to stringent inclusion and exclusion criteria, limiting its scope to studies that concurrently evaluated eGFR and mGFR within US cohorts. Therefore, the strictness of these criteria suggests caution in generalizing the findings to other contexts. Second, the review lacked extensive data on comorbidities and omitted measures of SDOH. Future studies should include racially diverse patient populations and adopt a broader approach that accounts for the associations of comorbidities and SDOH with kidney function and CKD-related outcomes.

## Conclusions

This systematic review and meta-analysis highlight the bias in race-based eGFR equations used for kidney disease diagnosis and management, emphasizing the need for race-independent eGFR equations. It also points out the limitations of using creatinine-based eGFR equations. The development of an accurate eGFR equation, independent of designated race, is emphasized as an important, yet initial, step toward equitable kidney health care. We must ensure that any shift to alternatives such as cystatin C–based eGFR equations does not lead us to prematurely believe that we have wholly addressed the issue of disparities in kidney health. The disproportionate burden of kidney failure on Black individuals demands a comprehensive, enduring effort that extends beyond diagnostic improvements. Truly mitigating racial disparities in CKD outcomes requires a multifaceted approach, which involves not only enhancing diagnostic tools but also addressing SDOH, confronting systemic racism, and implementing effective prevention and management strategies.

## Notes

Supplement 1. **eTable.** Search Strategy **eFigure 1.** PRISMA Flow Diagram **eFigure 2.** Accuracy in Different GFR Estimation Equations in Black Participants **eFigure 3.** Accuracy in Different GFR Estimation Equations in Non-Black Participants **eFigure 4.** Accuracy in GFR Estimation for Creatine-Based Equations in Subgroup Analysis **eFigure 5.** Accuracy in GFR Estimation for Creatinine-Cystatin C Equations in Subgroup Analysis **eFigure 6.** Accuracy in GFR Estimation for Cystatin C Alone Based Equations in Subgroup Analysis **eFigure 7.** Accuracy in GFR Estimation in Subgroup Analysis With Chronic Conditions **eFigure 8.** Bias in GFR Estimations in Subgroup Analysis With Chronic Conditions

Supplement 2.
Data Sharing Statement
