# Supplementary material for: Bias and Accuracy of Glomerular Filtration Rate Estimating Equations in the US: A Systematic Review and Meta-Analysis
Source: JAMA Netw Open. 2024 Mar 5;7(3):e241127. doi: 10.1001/jamanetworkopen.2024.1127 (PMC10915689; doi:10.1001/jamanetworkopen.2024.1127)
Supplement: Supplement 1. — eTable. Search Strategy eFigure 1. PRISMA Flow Diagram eFigure 2. Accuracy in Different GFR Estimation Equations in Black Participants eFigure 3. Accuracy in Different GFR Estimation Equations in Non-Black Participants eFigure 4. Accuracy in GFR Estimation for Creatine-Based Equations in Subgroup Analysis eFigure 5. Accuracy in GFR Estimation for Creatinine-Cystatin C Equations in Subgroup Analysis eFigure 6. Accuracy in GFR Estimation for Cystatin C Alone Based Equations in Subgroup Analysis eFigure 7. Accuracy in GFR Estimation in Subgroup Analysis With Chronic Conditions eFigure 8. Bias in GFR Estimations in Subgroup Analysis With Chronic Conditions [file jamanetwopen-e241127-s001.pdf]

Supplemental Online Content

Yan AF, Williams, Michelle Y, Shi Z, et al. Bias and accuracy of glomerular filtration rate estimation equations in the US: a systematic review and meta-analysis. *JAMA Netw. Open*. 2024;7(3):e241127. doi:10.1001/jamanetworkopen.2024.1127

eTable. Search Strategy

eFigure 1. PRISMA Flow Diagram

eFigure 2. Accuracy in Different GFR Estimation Equations in Black Participants

eFigure 3. Accuracy in Different GFR Estimation Equations in Non-Black Participants

eFigure 4. Accuracy in GFR Estimation for Creatine-Based Equations in Subgroup Analysis

eFigure 5. Accuracy in GFR Estimation for Creatinine-Cystatin C Equations in Subgroup Analysis

eFigure 6. Accuracy in GFR Estimation for Cystatin C Alone Based Equations in Subgroup Analysis

eFigure 7. Accuracy in GFR Estimation in Subgroup Analysis With Chronic Conditions

eFigure 8. Bias in GFR Estimations in Subgroup Analysis With Chronic Conditions

This supplemental material has been provided by the authors to give readers additional information about their work.

© 2024 Yan FY et al. *JAMA Network Open.*

eTable. Search Strategy

The final search numbers:

PubMed: 2238

Embase: 2261

Web of Science: 2158

ClinicalTrials.gov:6

Total results (before removing duplicates): 6663

Final PubMed Search

Date Searched: 1/20/2023

Set #	Concept	Syntax	Results
1	glomerular filtration rate	"glomerular filtration rate"[MeSH Terms] OR "glomerular filtration rate"[tw] OR GFR[tw] OR eGFR[tw] OR mGFR[tw]	140,151
2	algorithms	"Algorithms"[Mesh] OR "Algorithms"[tw] OR "Algorithms"[tw] OR “prediction model”[tw] OR “prediction models”[tw] OR equation[tw] OR equations[tw]	758,368
3	Development and validation	“Validation Study” [Publication Type] OR "Reproducibility of Results"[Mesh] OR reliability[tw] OR bias [tw] OR validation[tw] OR valid[tw] OR validity[tw] OR accuracy[tw]	1,752,454
4	Combining topics	#1 AND #2 AND #3	2,238

Full Search:

(("glomerular filtration rate"[MeSH Terms] OR "glomerular filtration rate"[tw] OR GFR[tw] OR eGFR[tw] OR mGFR[tw]) AND ("Algorithms"[Mesh]] OR "Algorithms"[tw] OR "Algorithms"[tw] OR "prediction model"[tw] OR "prediction models"[tw] OR equation[tw] OR equations[tw])) AND ("Validation Study" [Publication Type] OR "Reproducibility of Results"[Mesh] OR reliability[tw] OR bias [tw] OR validation[tw] OR validity[tw] OR accuracy[tw])

© 2024 Yan FY et al. *JAMA Network Open.*

Final Embase Search

Date Searched: 1/20/2023

Set #	Concept	Syntax	Results
1	glomerular filtration rate	'estimated glomerular filtration rate'/exp OR 'glomerular filtration rate':ti,ab,kw OR GFR:ti,ab,kw OR eGFR:ti,ab,kw OR mGFR:ti,ab,kw	233,436
2	algorithms	'algorithm'/exp OR 'Algorithms':ti,ab,kw OR 'Algorithms':ti,ab,kw OR 'prediction model':ti,ab,kw OR 'prediction models':ti,ab,kw OR equation:ti,ab,kw OR equations:ti,ab,kw	941,724
3	Development and validation	'validation study'/exp OR 'reproducibility'/exp OR reliability:ti,ab,kw OR reproducibility:ti,ab,kw OR validation:ti,ab,kw OR valid:ti,ab,kw OR validity:ti,ab,kw OR accuracy:ti,ab,kw OR bias:ti,ab,kw	2,111,313
4	Combining topics	#1 AND #2 AND #3	3,652
5	Removing conference abstracts	#4 NOT ([conference abstract]/lim OR [conference review]/lim)	2,261

© 2024 Yan FY et al. *JAMA Network Open.*

Final Web of Science Search

Date Searched: 1/20/2023

Set #	Concept	Syntax	Results
1	glomerular filtration rate	TS=("glomerular filtration rate" OR GFR OR eGFR OR mGFR)	133,253
2	algorithms	TS=("Algorithms" OR "Algorithms" OR "prediction model" OR "prediction models" OR equation OR equations)	2,261,718
3	Development and validation	TS=(reliability OR reproducibility OR validation OR valid OR validity OR accuracy OR bias OR derivation)	3,102,272
4	Combining topics	#1 AND #2 AND #3	3,652
5	Exclude conference abstracts	#4 excluding document type: conference abstract	2,158

Final ClinicalTrials.gov Search

Date Searched: 1/20/2023

6 Results:

(reliability OR reproducibility OR validation OR valid OR validity OR accuracy OR accurate OR derivation) AND ("glomerular filtration rate" OR GFR OR eGFR OR mGFR) AND ("Algorithms" OR "Algorithms" OR "prediction model" OR "prediction models" OR equation OR equations)

© 2024 Yan FY et al. *JAMA Network Open.*

eFigure 1. PRISMA Flow Diagram

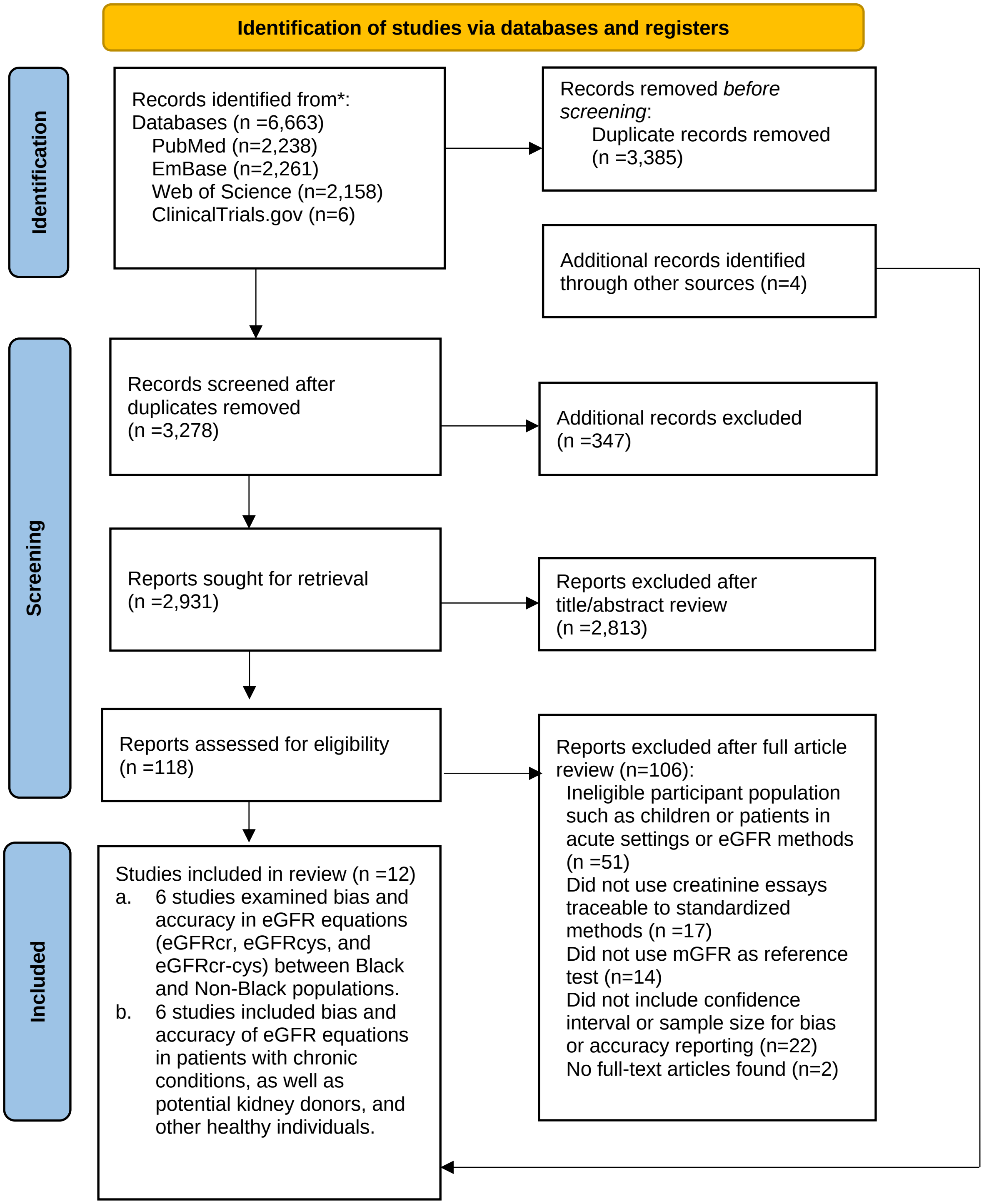

© 2024 Yan FY et al. *JAMA Network Open.*

eFigure 2. Accuracy in Different GFR Estimation Equations in Black Participants

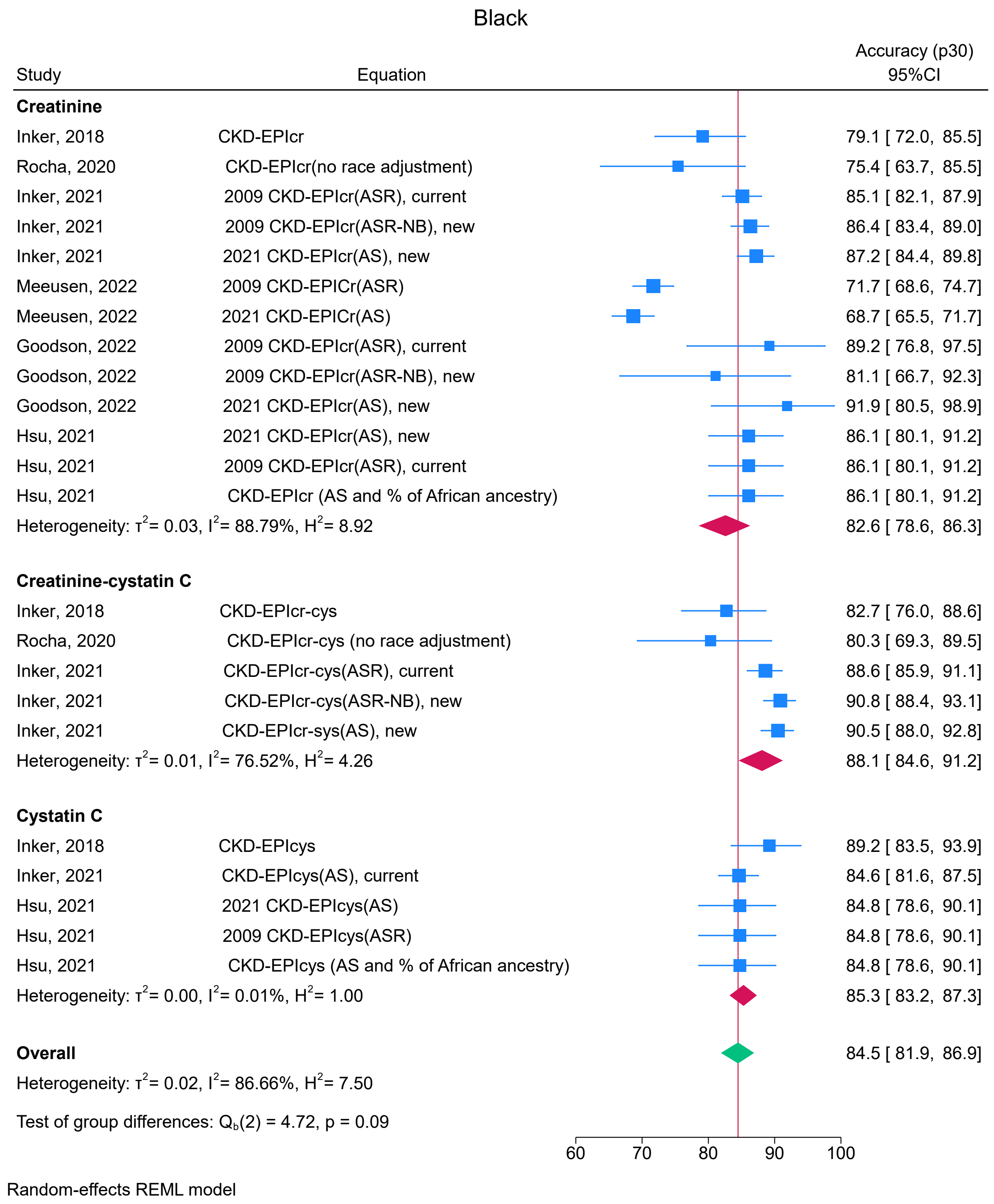

The Chronic Kidney Disease Epidemiology Collaboration (CKD-EPI) GFR estimating equations are referred to by the filtration marker or markers (serum creatinine [cr], cystatin C [cys]), and the combined cr-cys) and the demographic factors (age, sex, and race [ASR], or age and sex [AS] that were used in their development. ASR-Non-Black [NB] refers to ASR equations that were fit with a race term but in which the Black race coefficient was removed for computing of eGFR. Accuracy is assessed by P30, the percentage of people in a data set whose eGFR values are within 30% of measured GFR values. P30 values of 90% or higher indicate high accuracy.

eFigure 3. Accuracy in Different GFR Estimation Equations in Non-Black Participants

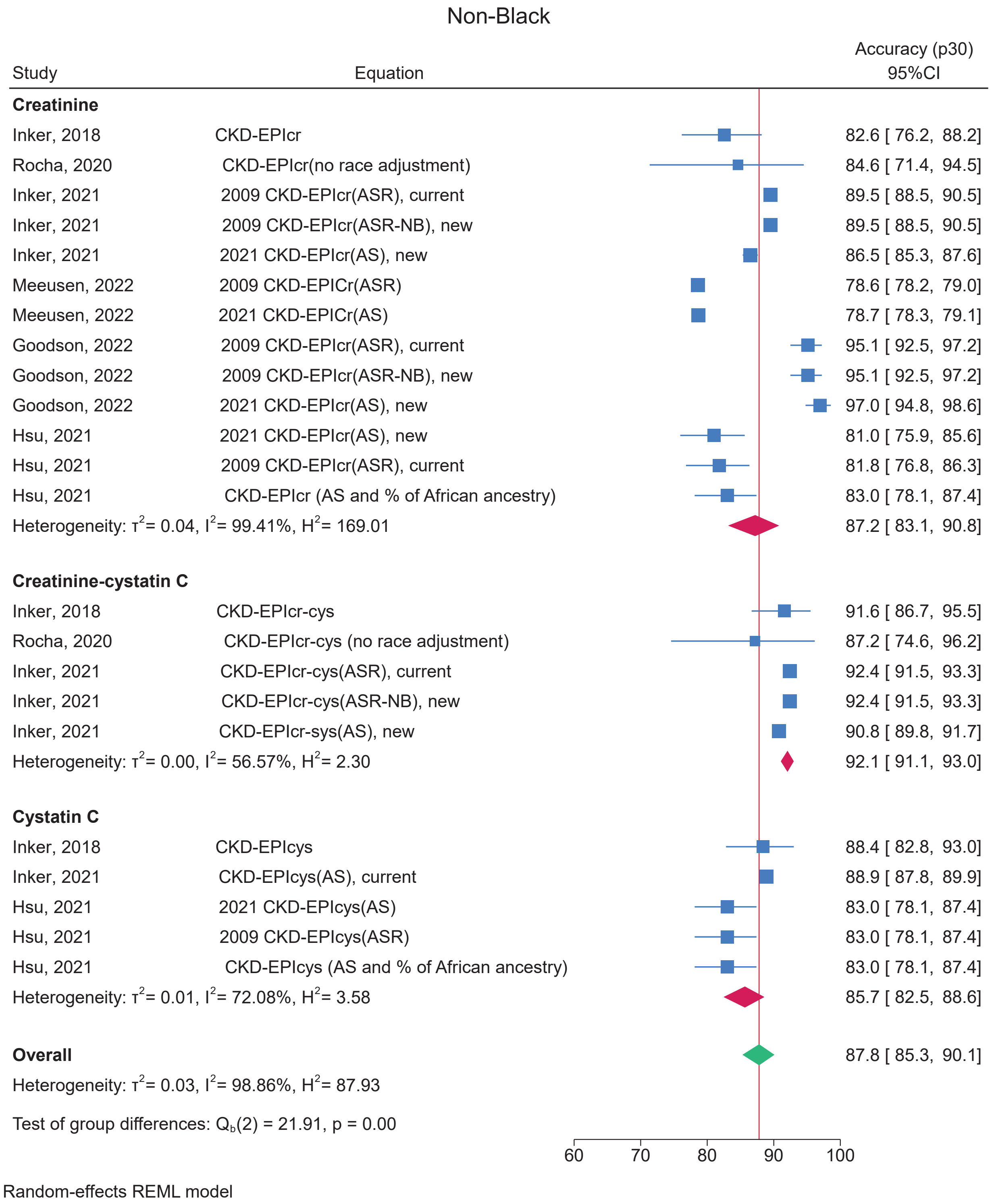

The Chronic Kidney Disease Epidemiology Collaboration (CKD-EPI) GFR estimating equations are referred to by the filtration marker or markers (serum creatinine [cr], cystatin C [cys]), and the combined cr-cys) and the demographic factors (age, sex, and race [ASR], or age and sex [AS] that were used in their development. ASR-Non-Black [NB] refers to ASR equations that were fit with a race term but in which the Black race coefficient was removed for computing of eGFR. Accuracy is assessed by P30, the percentage of people in a data set whose eGFR values are within 30% of measured GFR values. P30 values of 90% or higher indicate high accuracy.

eFigure 4. Accuracy in GFR Estimation for Creatine-Based Equations in Subgroup Analysis

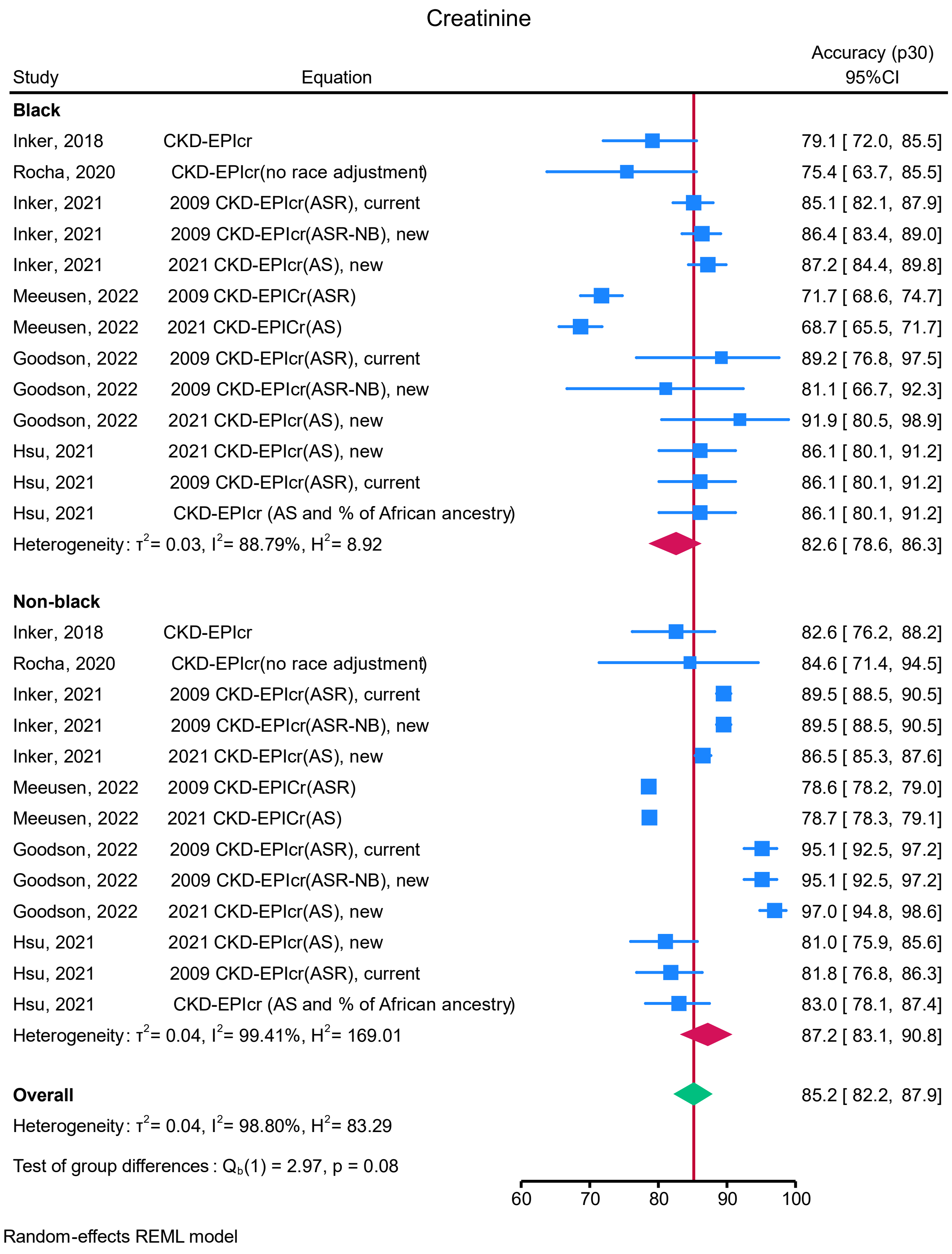

© 2024 Yan FY et al. *JAMA Network Open.*

eFigure 5. Accuracy in GFR Estimation for Creatinine-Cystatin C Equations in Subgroup Analysis

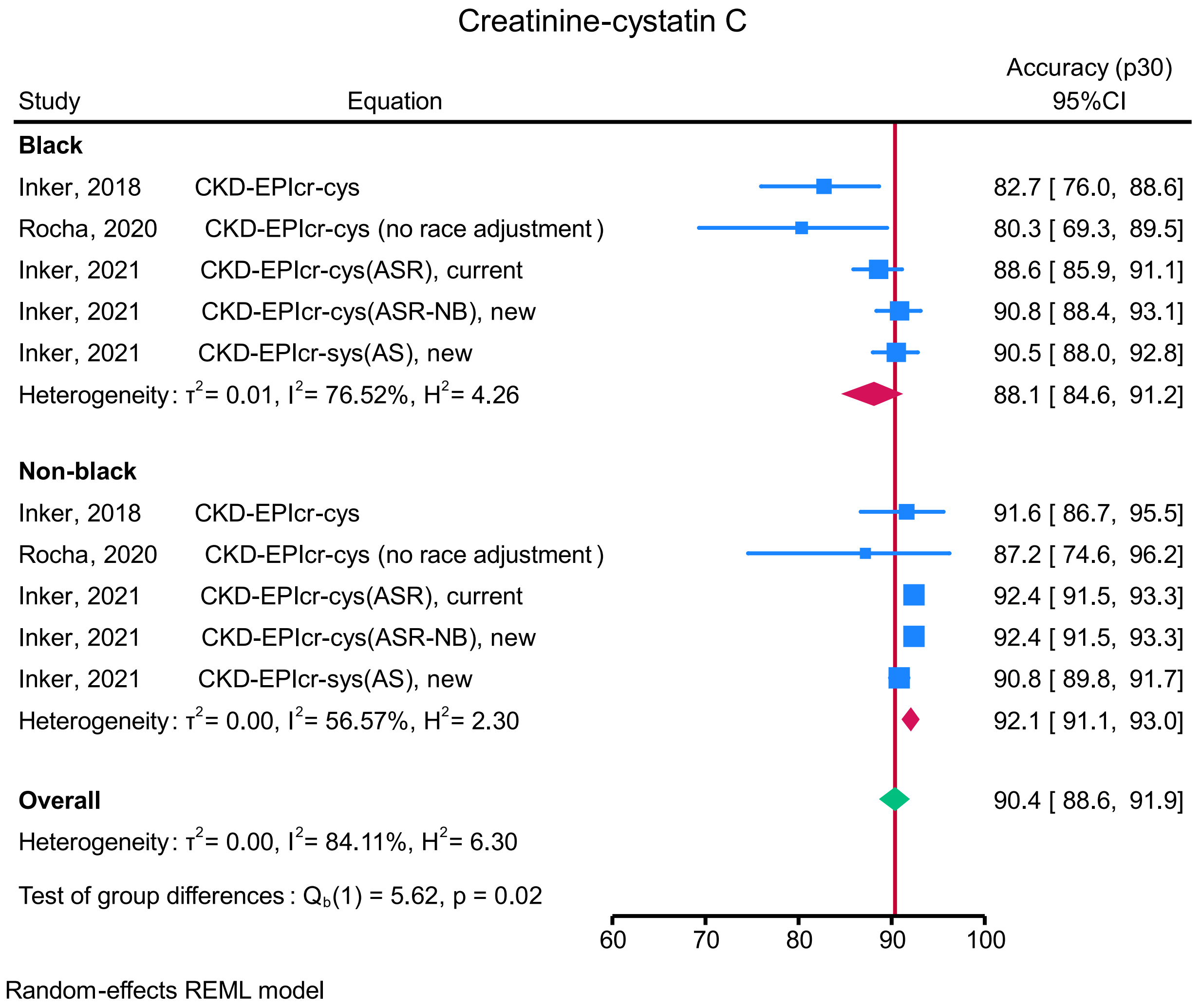

© 2024 Yan FY et al. *JAMA Network Open.*

eFigure 6. Accuracy in GFR Estimation for Cystatin C Alone Based Equations in Subgroup Analysis

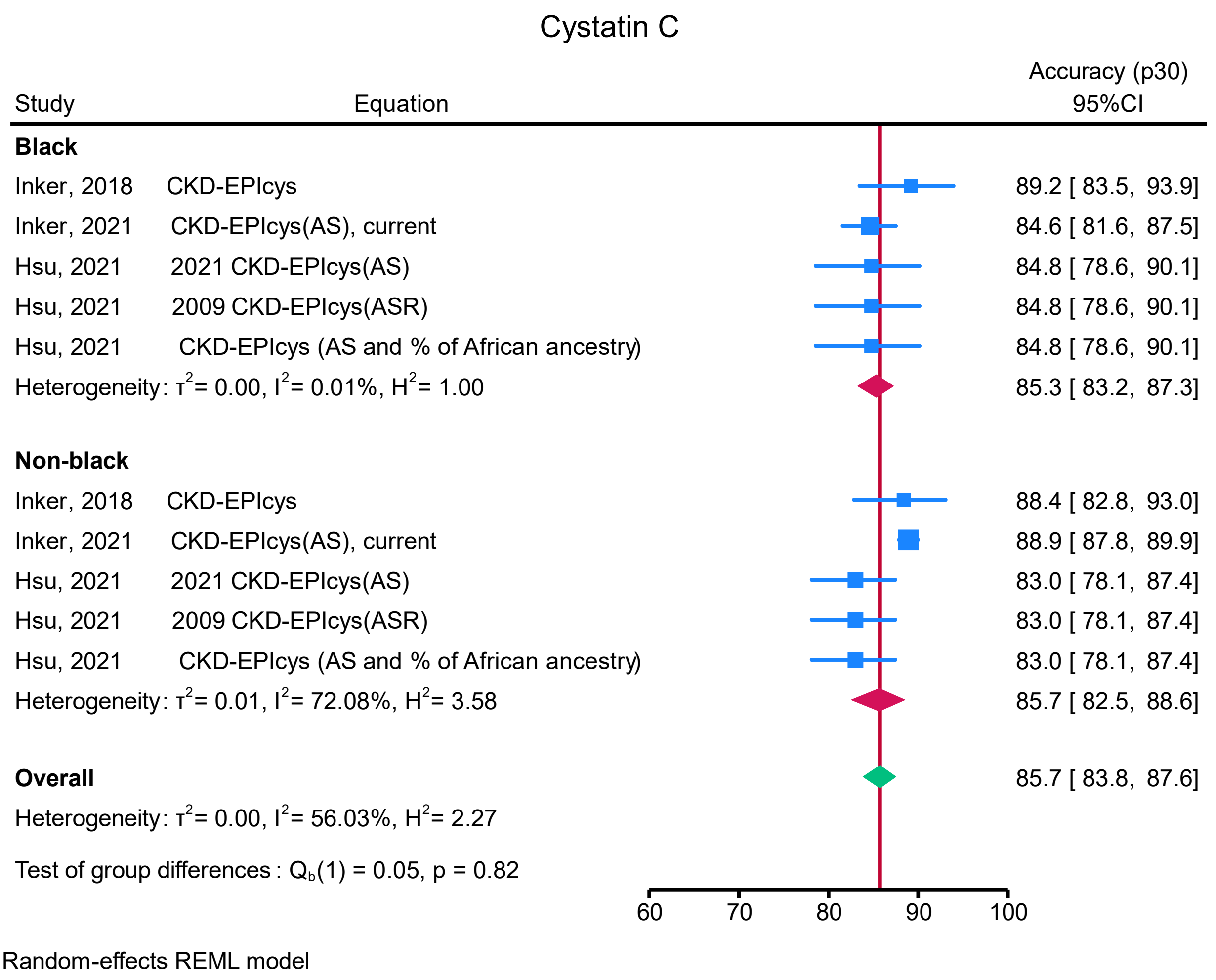

The Chronic Kidney Disease Epidemiology Collaboration (CKD-EPI) GFR estimating equations are referred to by the filtration marker or markers (serum creatinine [cr], cystatin C [cys]), and the combined cr-cys) and the demographic factors (age, sex, and race [ASR], or age and sex [AS] that were used in their development. ASR-Non-Black [NB] refers to ASR equations that were fit with a race term but in which the Black race coefficient was removed for computing of eGFR. Accuracy is assessed by P30, the percentage of people in a dataset whose eGFR values are within 30% of measured GFR values. P30 values of 90% or higher indicate high accuracy.

© 2024 Yan FY et al. *JAMA Network Open.*

eFigure 7. Accuracy in GFR Estimation in Subgroup Analysis With Chronic Conditions

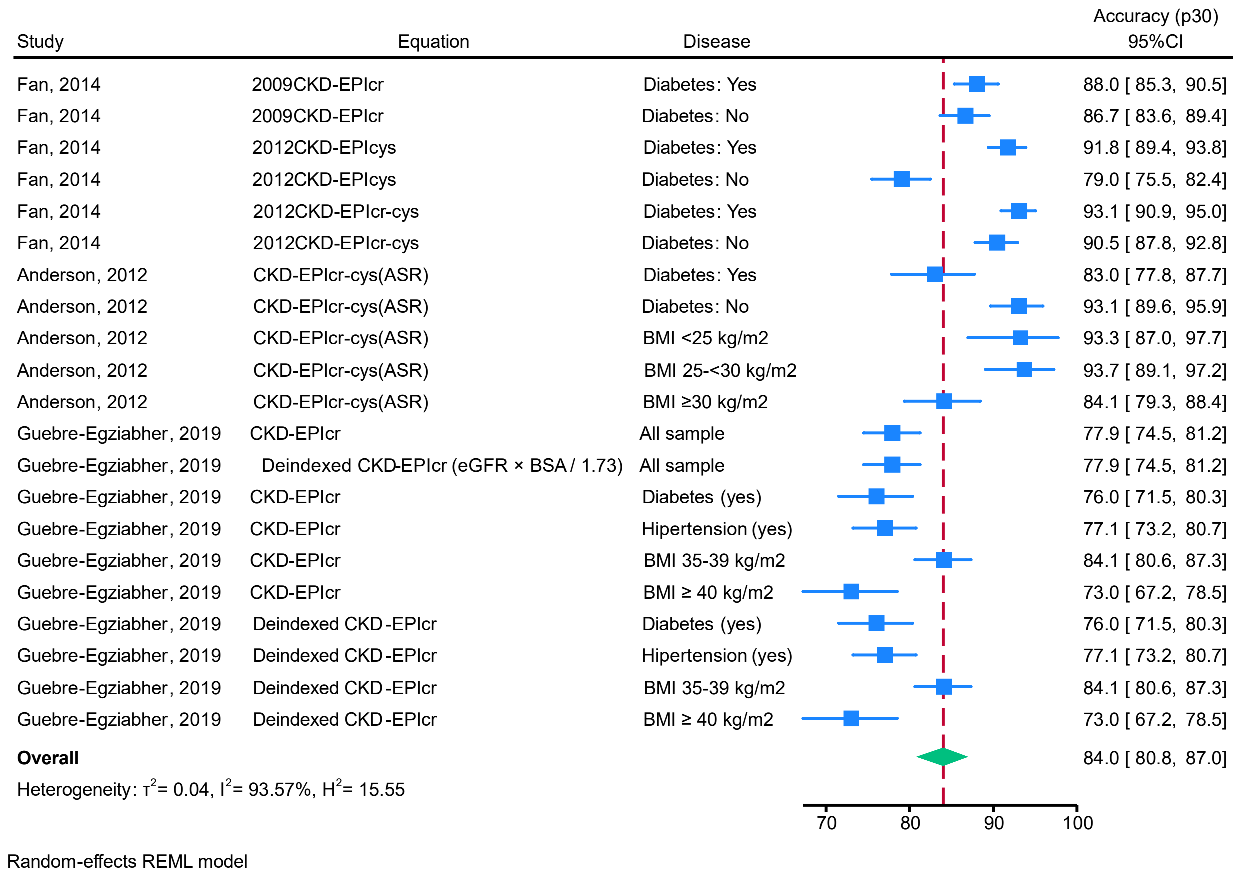

© 2024 Yan FY et al. *JAMA Network Open.*

eFigure 8. Bias in GFR Estimations in Subgroup Analysis With Chronic Conditions

Bias

Study	Equation	disease	Bias 95%CI
Aggarwal, 2012	CKD-EPICr	mean difference: mGFR-eGFR (Normal (BMI < 30)	6.5 [3.8, 9.2]
Aggarwal, 2012	CKD-EPICr	BMI 30–35	-3.0 [-6.9, 0.9]
Aggarwal, 2012	CKD-EPICr	BMI > 35	-19.2 [-23.4, -14.9]
Anderson, 2012	CKD-EPIcr-cys(ASR)	Median difference: median mGFR – eGFR Diabetes (yes)	0.0 [-5.5, 5.5]
Anderson, 2012	CKD-EPIcr-cys(ASR)	Diabetes (no)	1.0 [-4.5, 6.5]
Anderson, 2012	CKD-EPIcr-cys(ASR)	BMI <25 kg/m2	-2.0 [-6.0, 2.0]
Anderson, 2012	CKD-EPIcr-cys(ASR)	BMI 25-<30 kg/m2	2.0 [-3.5, 7.5]
Anderson, 2012	CKD-EPIcr-cys(ASR)	BMI ≥30 kg/m2	0.0 [-6.0, 6.0]
Fan, 2014	CKD-EPIcr	mean value of (mGFR – eGFR) (overall sample)	5.3 [4.5, 6.1]
Fan, 2014	CKD-EPIcr	mGFRcr categories, ≥90	14.3 [12.7, 15.9]
Fan, 2014	CKD-EPIcr	mGFRcr categories, 60–89	3.5 [1.6, 5.3]
Fan, 2014	CKD-EPIcr	mGFRcr categories, 30–59	0.3 [-0.8, 1.4]
Fan, 2014	CKD-EPIcr	mGFRcr categories, <30	-1.0 [-1.8, -0.2]
Fan, 2014	CKD-EPIcys	Overall sample	5.2 [4.4, 6.0]
Fan, 2014	CKD-EPIcys	mGFRcr categories, ≥90	14.0 [12.4, 15.6]
Fan, 2014	CKD-EPIcys	mGFRcr categories, 60–89	4.2 [2.4, 6.0]
Fan, 2014	CKD-EPIcys	mGFRcr categories, 30–59	0.7 [-0.3, 1.7]
Fan, 2014	CKD-EPIcys	mGFRcr categories, <30	-2.4 [-3.1, -1.7]
Fan, 2014	CKD-EPIcr-cys	Overall sample	5.4 [4.8, 6.1]
Fan, 2014	CKD-EPIcr-cys	mGFRcr categories, ≥90	13.1 [11.6, 14.6]
Fan, 2014	CKD-EPIcr-cys	mGFRcr categories, 60–89	4.4 [3.0, 5.8]
Fan, 2014	CKD-EPIcr-cys	mGFRcr categories, 30–59	1.4 [0.6, 2.2]
Fan, 2014	CKD-EPIcr-cys	mGFRcr categories, <30	-0.9 [-1.5, -0.3]
Hingorani, 2015	2009 CKD-EPIcr	Mean difference: mGFR-eGFR	15.6 [9.8, 21.4]
Hingorani, 2015	2012 CKD-EPIcys (Inker, 2012)		1.1 [-5.8, 7.9]
Hingorani, 2015	2012 CKD-EPIcr-cys (Inker, 2012)		7.9 [2.7, 13.1]
Keddis, 2016	CKD-EPIcr		-0.8 [-1.5, -0.1]
Keddis, 2016	CKD-EPIcys		8.7 [8.1, 9.2]
Keddis, 2016	CKD-EPIcr-cys		5.0 [4.5, 5.5]
Guebre-Egziabher, 2019	CKD-EPIcr	Median difference: mGFR-eGFR (all sample)	-7.3 [-8.4, -6.2]
Guebre-Egziabher, 2019	Deindexed CKD-EPIcr (eGFR × BSA / 1.73)	All sample	-9.0 [-10.2, -7.8]
Guebre-Egziabher, 2019	CKD-EPIcr	Diabetes (yes)	-6.9 [-8.1, -5.8]
Guebre-Egziabher, 2019	CKD-EPIcr	Hipertension (yes)	-7.0 [-8.1, -6.0]
Guebre-Egziabher, 2019	CKD-EPIcr	BMI 35-39 kg/m2	-7.3 [-8.4, -6.2]
Guebre-Egziabher, 2019	CKD-EPIcr	BMI ≥ 40 kg/m2	-7.3 [-9.5, -5.1]
Guebre-Egziabher, 2019	Deindexed CKD-EPIcr	Diabetes (yes)	-8.3 [-9.9, -6.7]
Guebre-Egziabher, 2019	Deindexed CKD-EPIcr	Hipertension (yes)	-8.5 [-10.0, -7.0]
Guebre-Egziabher, 2019	Deindexed CKD-EPIcr	BMI 35-39 kg/m2	-8.8 [-10.1, -7.6]
Guebre-Egziabher, 2019	Deindexed CKD-EPIcr	BMI ≥ 40 kg/m2	-9.5 [-12.1, -6.9]
**Overall**			0.2 [-1.7, 2.1]

Heterogeneity: τ^2 = 35.06, I^2 = 99.09%, H^2 = 109.40

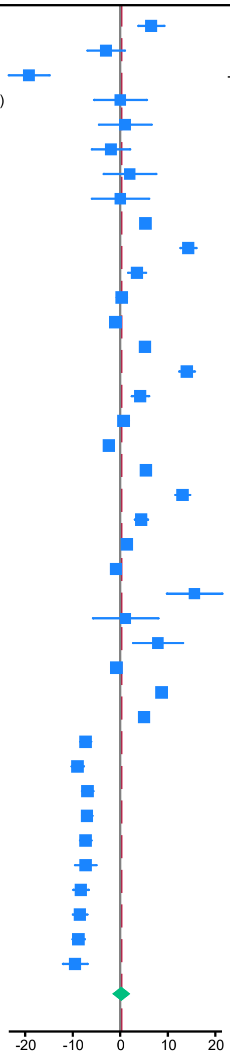

Random-effects DerSimonian–Laird model

The Chronic Kidney Disease Epidemiology Collaboration (CKD-EPI) GFR estimating equations are referred to by the filtration marker or markers (serum creatinine [cr], cystatin C [cys]), and the combined cr-cys) and the demographic factors (age, sex, and race [ASR], or age and sex [AS] that were used in their development. ASR-Non-Black [NB] refers to ASR equations that were fit with a race term but in which the Black race coefficient was removed for computing of eGFR. Bias is defined as the median difference between the measured Glomerular Filtration Rate (mGFR) and the estimated GFR (eGFR), calculated in milliliters per minute per 1.73 square meters (mGFR-eGFR) and accompanied by its 95% confidence interval (CI). A positive value of bias indicates that the eGFR tends to underestimate actual kidney function, while a negative bias value suggests an overestimation by eGFR. Accuracy is assessed by P30, the percentage of people in a dataset whose eGFR values are within 30% of measured GFR values. P30 values of 90% or higher indicate high accuracy.

© 2024 Yan FY et al. *JAMA Network Open.*
