# Supplementary material for: Bias and Accuracy of Glomerular Filtration Rate Estimating Equations in the US: A Systematic Review and Meta-Analysis
Source: JAMA Netw Open. 2024 Mar 5;7(3):e241127. doi: 10.1001/jamanetworkopen.2024.1127 (PMC10915689; doi:10.1001/jamanetworkopen.2024.1127)
Supplement: Supplement 2. — Data Sharing Statement [file jamanetwopen-e241127-s002.pdf]

Data Sharing Statement

Yan. Bias and Accuracy of Glomerular Filtration Rate Estimating Equations in the US. *JAMA Netw Open*. Published March 05, 2024. doi:10.1001/jamanetworkopen.2024.1127

Data
Data available: No
